# Metabolomics paves the way for improved drug target identification

**DOI:** 10.15252/msb.202210914

**Published:** 2022-02-23

**Authors:** Belinda B Garana, Nicholas A Graham

**Affiliations:** ^1^ Mork Family Department of Chemical Engineering and Materials Science University of Southern California Los Angeles CA USA; ^2^ Norris Comprehensive Cancer Center University of Southern California Los Angeles CA USA; ^3^ Leonard Davis School of Gerontology University of Southern California Los Angeles CA USA

**Keywords:** Metabolism, Methods & Resources, Pharmacology & Drug Discovery

## Abstract

Correctly identifying candidate drugs for protein targets is crucial for drug discovery. Despite the importance of this problem for the pharmaceutical industry, chemical screening remains a challenging task, and drug–target misidentification may contribute to failures in drug development. In their recent study, Sauer and colleagues (Holbrook‐Smith *et al*, 2022) demonstrate proof‐of‐concept for a new way to identify drug–target interactions using high‐throughput metabolomics, potentially paving the way towards a universal method for predicting drug–target relationships.

## Graphical abstract

Correctly identifying candidate drugs for protein targets is crucial for drug discovery. In their recent work, Sauer and colleagues (Holbrook‐Smith *et al*, 2022) demonstrate proof‐of‐concept for a new way to identify drug–target interactions using high‐throughput metabolomics.

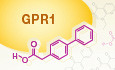

The advent of “omics” technologies including genomics, transcriptomics, proteomics, and metabolomics has presented an opportunity to discover drug candidates using systematic, global measurements of biomolecules. Over 20 years ago, early microarray profiling demonstrated that novel drug–target relationships could be identified by comparing the transcriptomic profiles of gene knockout and drug‐treated yeast (Hughes *et al*, 2000). Later efforts expanded this concept to mammalian cells treated with tens of thousands of drugs and other perturbagens (Subramanian *et al*, 2017). Proteomic approaches to drug target identification have also been successful at defining drug–target relationships, including thermal proteome profiling which exploits the increased resistance to heat‐induced protein unfolding that occurs upon drug binding (Savitski *et al*, 2014).

Notably, of all the “omic” layers, metabolomics is considered the closest to phenotype (Patti *et al*, 2012). Based on this idea, Holbrook‐Smith *et al* adopted a conceptually similar approach to that of Hughes *et al*, 2000. Specifically, Holbrook‐Smith *et al* compared the metabolomic profiles of the yeast *Saccharomyces cerevisiae* that had been subjected to either drug treatments or inducible overexpression of various intracellular and membrane‐bound proteins (Fig 1). If the metabolomic profiles following drug treatment and protein overexpression were highly correlated, then that drug was considered a candidate for the protein target. Testing this approach on membrane protein‐coding genes, Holbrook‐Smith *et al* identified and validated five novel antagonists for the G‐protein‐coupled receptor GPR1. Expanding to a larger set of 86 druggable genes, the authors further demonstrated that their method recovered true‐positive drug–target interactions with high sensitivity and specificity. Taken together, these results establish proof‐of‐concept for the identification of drug–target relationships using metabolomics.

**Figure 1:**
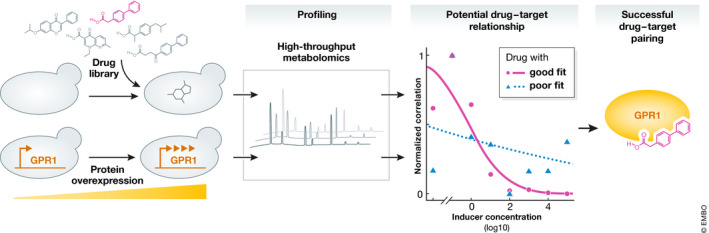
Drug target identification by high‐throughput metabolomics The metabolomic profiles of yeast treated with > 1,000 different drugs and yeast where a protein target had been inducibly overexpressed were collected by high‐throughput metabolomics. Candidate drugs were identified based on the similarity of the metabolomic profile of drug‐treated cells and that of cells with protein overexpression. This novel approach may represent a tool for universal prediction of drug–target relationships.

The key innovation that enables the approach of Holbrook‐Smith *et al* is flow injection time‐of‐flight mass spectrometry (FIA TOF MS), a high‐throughput metabolomic method which requires sample analysis time of < 1 min per sample (Fuhrer *et al*, 2011). This technique removes the bottleneck on the time required for MS sample analysis (~1 h per sample) in more traditional metabolomic approaches. With this method, the authors were able to profile thousands of conditions representing diverse drug treatments and protein overexpression conditions in a tractable manner, enabling the acquisition of a data set rich enough for candidate drug identification. Compared to traditional methods, this approach has several notable advantages. First, it leverages the ability of the metabolome to serve as the direct readout of cellular biochemistry. Secondly, by screening *in vivo*, drug candidates may be less susceptible to downstream development problems related to *in vivo* drug metabolism or the inability of drugs to reach intracellular targets. Lastly, this approach is amenable to membrane proteins, which are notoriously difficult to study biochemically yet represent many important drug targets.

Despite the advances of this project, significant hurdles remain before high‐throughput metabolomics becomes a tool for universal prediction of drug–target interactions. First, it will be important to show that this approach functions for drug discovery in mammalian cells. Although metabolic pathways are largely conserved between yeast and mammals, will differences in nutrient‐sensing mechanisms (González & Hall, 2017) and/or other pathways limit the applicability of this method in mammalian cells? Another question left unanswered is how media composition might impact drug target identification by metabolomics. In mammalian cells, media composition can significantly affect the response to drugs and genetic perturbations (Joly *et al*, 2021). As such, would the authors have found different results if their yeast had been cultured in a different media? Lastly, while FIA TOF MS enables rapid analysis of samples by omitting upstream chromatography, it is unable to resolve isomeric compounds. Are there drugs whose effects will be missed because of limitations in measuring critical metabolites? Similarly, there may be some druggable proteins whose effects on the metabolome are not captured by FIA TOF MS metabolomics. Answering these questions will be critical to realizing the potential of high‐throughput metabolomics for drug discovery.

In summary, the work of Holbrook‐Smith *et al* represents a significant step in expanding drug target discovery through metabolomics, the molecular “omics” layer closest to phenotype. Despite the billions of dollars poured into drug discovery, only roughly 14% of drug development programs result in FDA approval (Wong *et al*, 2019), although the usage of biomarkers based on stringent drug–target identification could improve the success rate of clinical trials. The success of Holbrook‐Smith *et al* suggests that high‐throughput metabolomics has broad potential to generate robust, whole‐genome predictions of drug–target interactions to improve drug development.
